# One-stage bilateral anterior bikini total hip replacement − experience of two cases

**DOI:** 10.1051/sicotj/2018030

**Published:** 2018-08-31

**Authors:** Ikram Nizam, Ashish V. Batra

**Affiliations:** Ozorthopaedics, 1356 High Street, Malvern, VIC 3144, Australia

**Keywords:** Bikini anterior hip replacement, Bilateral anterior hip replacement, One stage bilateral hip replacement, Enhanced recovery

## Abstract

Bilateral hip arthrosis is commonly seen and two-staged bilateral Total Hip Arthroplasty (THA) is a preferred choice of treatment due to the fear of complications associated with one-stage bilateral procedures. However, many studies now indicate that the treatment option should be patient specific. We report two cases of one-stage bilateral anterior THA. First, a 54-year-old male operated over a year ago and second a 59-year-old female operated 6 weeks ago. Both presented with a long history of bilateral grade IV hip arthritis causing them severe pain, compromised gait and disturbed sleep. Post-surgery, they were mobilized within few hours after surgery as part of our enhanced recovery programme. There was a drastic increase in their Harris Hip Scores from 34.5 in case 1 and 19.2 in case 2 to 100 for both hips in both patients. They reported excellent recovery, regained normal gait pattern, returned quickly to their routine lifestyle and felt it was an economical option. We conclude that one-stage bilateral bikini anterior THA is a preferred surgical option for patients with bilateral hip arthritis that are medically fit.

## Introduction

Total Hip Arthroplasty (THA) has become a preferred treatment option for end stage arthrosis helping patients to regain independence and achieve a good quality of life. The number of THAs performed are expected to increase over the coming years with many requiring a contralateral hip arthroplasty after the index procedure [1].

There is no clear clinical consensus in the literature on which is better, between one-stage bilateral versus staged bilateral THA procedures. Some studies indicate higher incidence of complications after one-stage bilateral THA [2]. Heterotopic ossification, blood loss, higher prevalence of deep vein thrombosis, and greater risk of pulmonary complications are amongst the main reported complications post one-stage bilateral THA [3–5]. These results have been reported to be much better with improved anaesthetic and surgical techniques and postoperative care. Some of the benefits of bilateral one stage THA include more efficient use of resources, reduced hospitalization and shorter rehabilitation [4]. Further, the improvements in various elements of walking are reportedly higher in patients with bilateral THA than in those with unilateral staged replacement.

There are various surgical approaches used for THA, the most common being the posterior approach. However, with modern advancements and innovations, studies show that anterior hip arthroplasty claims to have less damage to muscles and hence quicker recovery with less pain, early return to routine activities and early mobilisation with improved gait [4,6,7].

We present two patients and report their recovery after bilateral THAs for end stage arthritis, in which one-stage bilateral anterior bikini hip arthroplasties were performed.

## Case 1

In October 2016, A 54-year-old male presented with bilateral crippling hip pain in the groin on both sides radiating to the front of the thigh with reduced mobility for almost 3 years and progressive worsening of symptoms. He had developed a significant limp preventing him from walking and performing routine activities independently with disturbed sleep.

**Figure 1:**
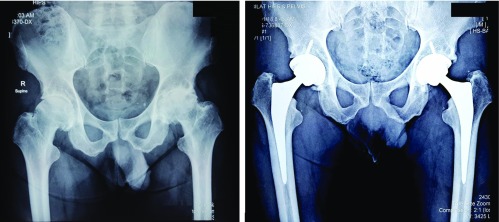
Pre-operative AP X-Ray pelvis with both hip joints (Case 1) showing grade IV OA both hip joints and one year follow-up post-operative X-ray after bilateral uncemented bikini hip replacement showing well aligned acetabular and femoral prosthesis in situ. (Case 1). During surgery the stem was prepared first followed by the cup. Prosthesis details: Acetabular shell: R3 three hole HA acetabular shell (Smith and Nephew Memphis Tennessee). Acetabular liner: R3 XLPE (Smith and Nephew). PolarStem femoral stem (uncemented − Smith and Nephew AG, Baar, Switzerland), femoral head: oxinium femoral head (Smith and Nephew Memphis Tennessee). The total operative time was 2 h and 15 min.

On examination, he had a bilateral stiff hip, antalgic gait with a BMI of 34.1. Both hips had very limited range of motion (Table 1). He had a poor Harris Hip Score of 34.2 in the left hip and 34.3 in the right hip. Anteroposterior X-ray of the pelvis with both hip joints showed severe bone-on-bone arthritis (Figure 1) in both the hip joints. He elected to undergo simultaneous bilateral soft tissue sparing bikini anterior hip replacements described previously by the senior surgeon [8]. The left hip was operated first followed by right with a surgical time of 135 minutes total.

**Table 1:** Range of movements preoperative and postoperative for both hip for both case one and case two.

Particulars	Patient 1				Patient 2			
	(Male, 54 yrs)				(Female, 59 yrs)			
		
	Left hip		Right hip		Left hip		Right hip	
				
	Pre-operative	Post-operative	Pre-operative	Post-operative	Pre-operative	Post-operative	Pre-operative	Post-operative
***Range of movements***								
Fixed Flexion Deformity	20°	0°	10°	0°	25°	0°	20°	0°
Flexion	80°	120°	90°	120°	90°	120°	90°	120°
Abduction	25°	30°	25°	30°	20°	30°	15°	30°
Adduction	10°	25°	10°	25°	10°	30°	5°	30°
Internal Rotation	<10°	30°	0°	30°	5°	30°	3°	30°
External Rotation	25°	30°	20°	30°	10°	30°	10°	30°
								
HARRIS HIP SCORE	34.2	100	34.3	100	19.5	100	19.4	100

Post-operatively, mechanical thromboprohpylaxis was used for 24 h followed by oral aspirin 300 mg with nexium for 6 weeks. He was mobilized within few hours after surgery as part of our enhanced recovery programme. He started walking with the aid of a walking frame and even managed a dozen steps unaided the same day. He was discharged on the 2nd post-operative day. He started on his exercise bike Day 5 post op and resumed driving on the sixth day onwards as he was very mobile with a single crutch mainly for safety and not on any narcotic analgesia. He felt very confident and comfortable and had no issues driving.

On day 9 post-op, he was back at work doing light duties and clerical activities by which time he was mobile with pain free hip movements and without any mechanical dysfunction. At the 6 week mark he had a well healed surgical scar with no swelling and walked in without a limp. He was followed up regularly at 3 months and 12 months post-operatively. His last follow up was 16 months post-surgery by which time he was having no issues in either hip joints and was able to do all his day to day activities, with a highly improved quality of life. His X-rays (Figure 1) were showing well aligned acetabular and femoral prosthesis insitu on both sides. His post-operative Harris Hip score was excellent, for both hips being 100.

## Case 2

In October 2017, a 59-year-old female presented with severe pain in both her hips over the preceding 3 years. Most of her pain was localized to her groin, right worse than the left. Over the preceding few months her pain increased to a level where she couldn't perform her normal daily activities with disturbed sleep at night. Along with this pain, she started developing a noticeable limp over 12 months. She used two crutches to support herself with a walking distance of only 30–50 m. Her quality of life was severely compromised and taking strong analgesics including opiods.

**Figure 2:**
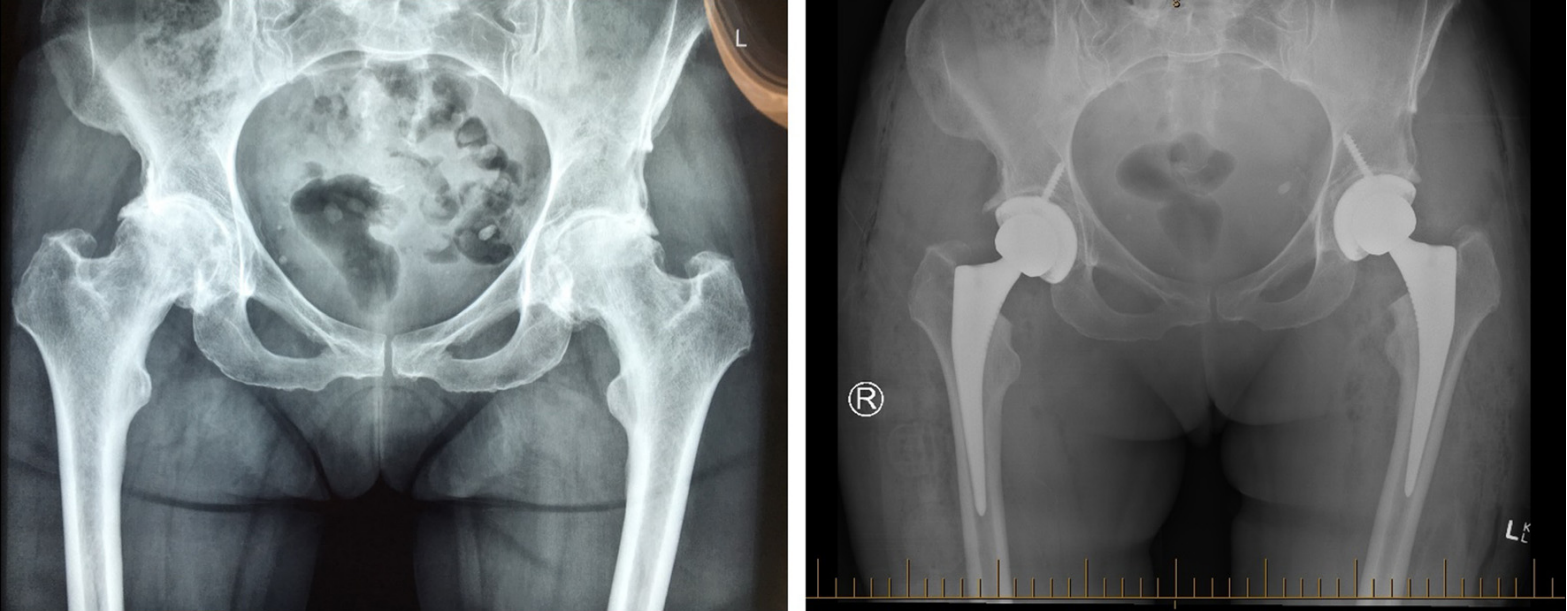
Pre-operative AP X-Ray pelvis with both hip joints (Case 2) showing grade IV OA both hip joints and post-operative X-ray after bilateral uncemented bikini hip replacement showing well aligned acetabular and femoral prosthesis in situ (Case 2).

On examination, she walked with a bilateral stiff hip antalgic gait with a BMI of 24.1. Both her hips had very limited range of motion (Table 1). She had a poor Harris Hip Score of 19.5 for the left hip and 19.4 for the right hip. Her X-rays revealed, severe bone on bone osteoarthritic changes bilaterally (Figure 2). She underwent one stage bilateral anterior hip replacement.

**Figure 3:**
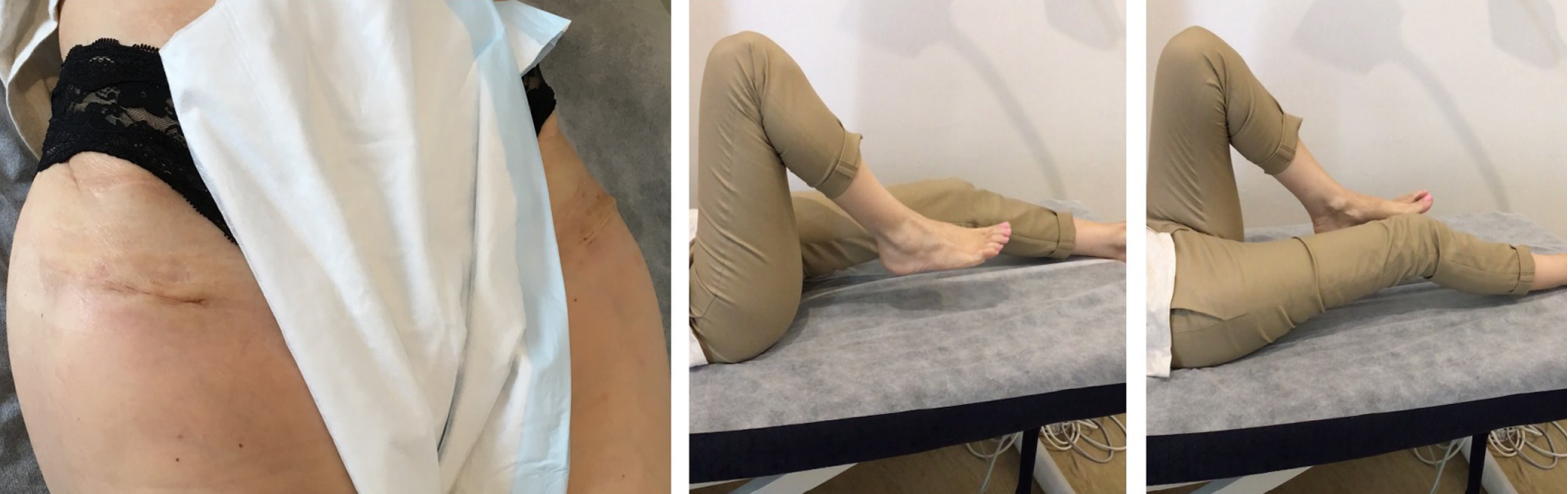
Post-operative hip flexions and scars healing for right and left hip joints at 2 weeks (Case 2).

The same post-operative recovery protocol was followed as in the first case. She went to rehab on day 3 as she lived alone. At her 2-week post-operative visit, she was doing excellent and hardly experienced any pain. She had a well healed scar with hip flexion beyond 90° in both hips (Figure 3). She commenced driving within 2 weeks. On her 6-week post-operative visit, her Harris Hip Score was 100 for both hips.

The prosthesis used for both the cases was polar stem (uncemented − Smith and Nephew AG, Baar, Switzerland), Oxinium femoral head (Smith and Nephew Memphis Tennessee) R3 three hole HA acetabular shell (Smith and Nephew Memphis Tennessee) and acetabular liner R3 XLPE (Smith and Nephew).

## Discussion

In literature, there are various cases reported of bilateral total hip replacement using posterior or lateral approaches. This report highlights the advantage of soft tissue sparing technique of the bikini hip replacement which enables patients to achieve rapid recovery with early return to activities including safe driving within days after bilateral anterior hip replacements. There are various studies that show the clinical benefits and durable results of direct anterior (DAA) hip replacements, but there isn't enough data detailing patient specific results and satisfaction when comparing one staged vs two staged THA. Moreover, there is even less data comparing bilateral direct anterior hip arthroplasty in the one-stage vs two-stage settings.

A study by Power et al. [9] reported that simultaneous bilateral total hip arthroplasty is a valuable therapeutic option in appropriately selected patients wit bilateral degenerative hip disease. We believe that the opinion on doing one hip first, followed by second or to do bilateral hip at a time cannot be generalized. In these specific cases we felt that doing one-staged THAs were better, since both hips were equally arthritic, and replacing one hip would not have achieved the results that we otherwise did, since the gaits would have still been compromised after unilateral surgery. Secondary benefits reported from studies included less use of resources, quicker recovery, less hospital stay and hence more economical. In a study by Rolfson et al. [10] cost data showed that in a theoretical model, comparing one- and two-staged procedures showed a 24% reduction in hospital and sick-leave costs in favour of the one-stage bilateral THA in healthy patients. Both our patients returned to work within 4 weeks.

The Harris hip scores of the patients improved from a poor score of 34.2 in case one and 19.5 in case two to an excellent score of 100 in both cases within 6 weeks. This muscle/soft tissue sparing approach enabled patients to get back to routine activities early, with minimal restrictions and they were driving within 6–12 days after surgery. There were no complications in either cases.

We would recommend bilateral anterior THA in cases with severe bilateral bone on bone arthritis in patients who are medically fit, motivated and under 70 years of age after detailed informed consent.

## Conflict of interest

The authors declare that they have no conflicts of interest in relation to this article.

## References

[1] Alfaro-Adrian J, Bayona F, Rech JA, Murray DW (1999) One- or two-stage bilateral total hip replacement. J Arthroplasty 14(4), 439–445. 10428224 10.1016/s0883-5403(99)90099-2

[2] Berend ME, Ritter MA, Harty LD, Keating EM, Meding JB, Thong AE (2005) Simultaneous bilateral versus unilateral total hip arthroplasty an outcomes analysis. J Arthroplasty 20(4), 421–426. 16124956 10.1016/j.arth.2004.09.062

[3] Parvizi J, Tarity TD, Sheikh E. (2006) Bilateral total hip arthroplasty: one-stage versus two-stage procedures. Clin Orthop Relat Res 453, 137–141. 17312590 10.1097/01.blo.0000246529.14135.2b

[4] Bhan S, Pankaj A, Malhotra R (2006) One - or two-stage bilateral total hip arthroplasty: a prospective, randomised, controlled study in an Asian population. J Bone Joint Surg (Br) 88(3), 298–303. 16498000 10.1302/0301-620X.88B3.17048

[5] Trojani C, d'Ollonne T, Saragaglia D, Vielpeau C, Carles M, Prudhon J-L (2012) One-stage bilateral total hip arthroplasty: Functional outcomes and complications in 112 patients, Orthop & Traumatol: Surg & Res 98(6) S120–S123. 22939864 10.1016/j.otsr.2012.06.008

[6] Taunton MJ, Mason JB, Odum SM, Springer BD (2014) Direct anterior total hip arthroplasty yields more rapid voluntary cessation of all walking aids: a prospective, randomized clinical trial. J Arthroplasty 29, 169–172. 25007723 10.1016/j.arth.2014.03.051

[7] Martin CT, Pugely AJ, Gao Y, Clark CR (2013) A comparison of hospital length of stay and short-term morbidity between the anterior and the posterior approaches to total hip arthroplasty. J Arthroplasty 28, 849–854. 23489731 10.1016/j.arth.2012.10.029

[8] Nizam I (2015) The bikini hip replacement − surgical technique preserving vessels and deep soft tissues in direct anterior approach hip replacement. J Orthop Res Physiother 1, 007.

[9] Power FR, Cawley DT, Curtin PD (2017) Simultaneous bilateral total hip arthroplasties in nonagenarians. Ir J Med Sci 186(4), 947–951. 28185060 10.1007/s11845-017-1572-5

[10] Rolfson O, Digas G, Herberts P, Karrholm J, Borgstrom F et al. (2014) One-stage bilateral total hip replacement is cost-saving. Orthop Muscul Syst 3, 175.

